# Management of genetic defects in breeding programs of species with high prolificacy

**DOI:** 10.1093/jas/skag100

**Published:** 2026-03-25

**Authors:** Silvia T Rodríguez-Ramilo, Isabelle Palhière, Jérôme Raoul, Jesús Fernández

**Affiliations:** GenPhySE, Université de Toulouse, INRAE, ENVT, Castanet-Tolosan 31326, France; GenPhySE, Université de Toulouse, INRAE, ENVT, Castanet-Tolosan 31326, France; GenPhySE, Université de Toulouse, INRAE, ENVT, Castanet-Tolosan 31326, France; Idele, Castanet-Tolosan, CS, 52637, 31321, France; Departamento de Mejora Genética Animal, INIA—CSIC, Madrid 28040, Spain

**Keywords:** selection scheme, genetic defect, genetic gain, genetic diversity, allele frequency

## Abstract

Advances in animal breeding have improved productivity but have led to concerns like reduced genetic diversity and the augmentation of the frequency of genetic defects. This increase of the frequency of genetic defects can be attributed to a hitchhiking effect or to genetic drift in populations with small effective population size. The aim of this study was to compare strict culling of carriers of a genetic defect with balanced selection strategies aimed at reducing the frequency of a genetic defect while maintaining genetic diversity and genetic gain in breeding programs of species with high prolificacy. A simulation-based approach was employed to model breeding programs considering different initial frequencies of a genetic defect, selection pressures, and correlations between the genetic defect and a productive trait. Results indicate that a balanced approach can effectively eliminate the defect with no severe consequences on the outcomes of the breeding program but a slight reduction in the genetic progress and the genetic diversity. A high initial allele frequency of the genetic defect and a correlation between the defect and the productive trait make it more complicated to eradicate the genetic defect.

## Teaser

Advances in animal breeding have enhanced productivity but have also raised concerns about decreasing genetic diversity and an increased frequency of genetic defects. This study highlights that, in species with high prolificacy, a balanced approach is effective in eliminating the genetic defect with only a slight risk of reducing genetic diversity and genetic progress in breeding programs.

## Introduction

In recent years, there has been a notable enhancement in animal productivity, primarily attributable to the implementation of effective breeding programs based on genetics. Nevertheless, while rigorous selection approaches contribute to improved performance and, thus, to higher profitability, they also pose a risk through the likely reduction of the effective population size (*N_e_*). This reduction in *N_e_* runs in parallel to a larger loss of genetic diversity (expressed, for example, as an increment of coancestry) and the increase in inbreeding. The drawbacks of both processes on the ability for responding to selection in the long term and the development of inbreeding depression are well known (Lynch and Walsh 1998). Indeed, considering the repercussions of climate change, preserving genetic diversity will be essential to ensure the resilience of populations and breeding programs (Berry et al. 2022). An additional problem connected to the selection process and the reduced *N_e_* is the possible rise of the frequency of monogenic genetic defects. Thus, besides the harmful effects of inbreeding on the general fitness of individuals, this rise of frequencies may be leading to the recurrent manifestation of Mendelian defects within the industry, which causes insightful effects on economic sustainability (Charlier et al. 2008; Schmidt et al. 2023; Cole et al. 2025).

The increase of the prevalence of monogenic defects within populations can be explained by three main mechanisms. First, the genetic hitchhiking effect can increase the frequency of deleterious alleles when they are in linkage disequilibrium with favorable alleles targeted by selection. This may occur because loci underlying selected traits and those responsible for genetic defects are in close physical proximity, resulting in their joint transmission during reproduction (Smith and Haigh 1974). The likelihood of this situation in polygenic traits is low; however, Quantitative Trait Loci (QTL, thereafter) explaining a relevant percentage of the genetic variation have been described in many species and traits, and, consequently, they cause a selection signature where the loci controlling the defect can be shifted to high frequencies. Second, if the deleterious allele has epistatic effects on the selected trait (improving its performance), individuals with high genetic value for the trait will probably carry deleterious alleles for the genetic defect. Finally, genetic drift associated with small *N_e_* can lead to elevated frequencies of genetic defects by driving random changes in allele frequencies (Caballero and Toro 2002). This effect is amplified when individuals carrying such defects contribute disproportionately to subsequent generations, as commonly observed with the extensive use of artificial insemination (Georges et al. 2019). Consequently, populations with reduced census size and/or *N_e_* are particularly susceptible to the accumulation of monogenic defects.

The detection and eradication of genetic defects has become a significant concern due to the increasingly appearance of defects that clearly impact animal welfare and carry substantial economic consequences (Ben Braiek et al. 2021). Several cases illustrate monogenic recessive defects in livestock populations, for example, in dairy cattle (Gozdek et al. 2024), sheep (Fabre et al. 2021), pig (Derks et al. 2019) and rabbit (Botha et al. 2014) populations and in aquaculture species (Colihueque and Araneda 2014). When breeders notice the existence of a genetic defect in their populations, the urgent aim is to remove the detrimental genetic information. A simple and immediate option is to conduct a pre-selection process, culling animals expressing the defect or known to be carriers of the deleterious allele (Hjortø et al. 2021). It must be recalled that many genetic defects (especially those lethal) are recessive (Berry and Spangler 2023), and, therefore, an individual must be homozygous for the deleterious allele to effectively express the genetic defect. The above procedure (ie, culling individuals) ensures that known genetic defects are absent from the commercial population and reassures customers about the quality of the final product. However, as the number of identified genetic defects increases, the proportion of defect-free animals diminishes significantly, with the corresponding reduction of candidates to selection and the selection differential, which leads to significant reductions in the achieved genetic gain. For example, intensive selection pressure applied in certain sheep breeds to favor genotypes conferring genetic resistance to scrapie resulted in a consequential decline in the rate of genetic improvement for economically important productive traits (Palhière et al. 2004). Moreover, as a side effect, the exclusive utilization of these defect-free individuals is likely to result in the emergence of new defects in subsequent generations if they are carriers of still unknown/undetected alleles controlling genetic defects (Georges et al. 2019). Nevertheless, the evolution of the frequencies of the deleterious alleles is not the same when breeding homozygous or heterozygous animals, and, therefore, the condition of the candidates to selection for the defect should be considered. This can be accounted for through the concept of “gene content” (Falconer and Mackay 1996; Forneris et al. 2015) that is just the number of copies of the deleterious allele in the individual.

Consequently, dealing with populations harboring individuals with genetic defects require specialized breeding programs designed to effectively reduce the frequency of these defects while simultaneously maximizing genetic progress (and also restricting inbreeding to an acceptable level). Several examples exist in literature proposing strategies to tackle this complex task. These approaches can be assembled in methodologies dealing with 1) mating strategies, 2) selection indices, and 3) sequential mate allocation schemes. Regarding the first type of approaches, one straightforward strategy involves avoiding matings between carriers (Charlier et al. 2008; Derks and Steensma 2021), thereby preventing the creation of deleterious homozygous offspring. Linear programming can also be employed to optimize the economic value of mating decisions (Bengtsson et al. 2023). In the second group of methodologies, it is well known that selection indices are primarily designed for ranking animals for selection purposes, but they can be also employed to manage genetic defects (eg, Boichard et al. 2016; Segelke et al. 2016; personal communication). Concerning the last type of approaches, Pryce et al. (2012) demonstrated the effectiveness of a sequential mate allocation scheme to reduce pedigree and genomic inbreeding, which also decreases the frequency of homozygous individuals, and consequently, the expression of recessive alleles. Expanding upon this work, Cole (2015) also proposed a sequential mate allocation scheme that penalized the average of the parents’ TBV of each potential mating pair for both inbreeding effects and the potential losses resulting from matings between carriers. Their results revealed a reduction in undesirable allele frequencies, a slightly lower genetic gain, and minimal impact on inbreeding rates. Cole’s (2015) method has been applied to Montbéliarde breed (Bérodier et al. 2021).

Several of the aforementioned alternatives for managing genetic defects have been evaluated using simulated and real data from species exhibiting low prolificacy (for a review in dairy cattle, see eg, Cole et al. 2025). However, to our knowledge, the effectiveness of managing genetic defects, or implementing strict culling of animals expressing the defect or identified as carriers of the deleterious allele, remains to be determined in species characterized by high prolificacy and, occasionally, with discrete generations, such as pigs (Castro Dias Cuyabano et al. 2019; Zhao et al. 2021; Sharif-Islam et al. 2024), rabbits (Garreau et al. 2005; Blasco et al. 2017), poultry (Mehrabani-Yeganeh et al. 1999; König et al. 2010; Harrison et al. 2023), or aquaculture species (Skaarud et al. 2014; Fernández et al. 2021). In the context of this study and following the definitions from FAO guidelines (FAO 2013), high-prolificacy species are defined as those characterized by large family sizes, such as pigs, rabbits, guinea pigs, avian species, and aquaculture species. In contrast, low-prolificacy species, such as cattle, sheep, horses, or camelids, are characterized by small family sizes, which makes the management of genetic defects more restrictive.

This study aimed to compare the strategy of strict culling of carriers of genetic defects against a balanced selection accounting for genetic gain and the reduction of the presence of the genetic defect in a high-prolificacy species. The evaluation of the performance of each strategy was made on the achieved genetic gain for the target trait, the amount of genetic diversity kept, and the evolution of the allelic frequencies in the locus controlling the defect. Computer simulations where run to obtain the results for the different scenarios.

## Materials and methods

### Base population: individuals, genomes, quantitative trait and genetic defect

In a first step, a base population in mutation-drift equilibrium was established, following the approach outlined by Morales-González et al. (2021). Over 10,000 discrete generations, random mating was simulated for a population consisting of 100 individuals (50 males and 50 females). Each generation, sires and dams were randomly selected with replacement, and population size was kept constant across generations. The genome comprised 20 chromosomes, each with a length of 1 Morgan. Two types of biallelic loci were simulated: SNP loci to be used for genomic evaluations during the selection step performed on the base population and non-marker loci used to test the consequences of each management method on the diversity maintained. Each chromosome had 500,000 SNPs and 500,000 non-marker loci. Initially, the allelic frequencies for all loci were 0.5. The mutation rate per locus and generation (*m*) was set at 2.5 × 10^−6^ for all loci, with the number of new mutations per generation sampled from a Poisson distribution with mean *2Nn_c_mn_l_*, where *N* is the population size, *n_c_* is the number of chromosomes, and *n_l_* is the total number of loci per chromosome. Mutations were randomly distributed across individuals, chromosomes, and loci, switching allele 1 to allele 0 and vice versa. During gamete formation, the number of crossovers per chromosome followed a Poisson distribution with a mean of one and were randomly distributed without interference. After 10,000 discrete generations, the expected heterozygosity (*H_e_*) stabilized at both types of loci, indicating mutation-drift equilibrium.

After the establishment of the mutation-drift equilibrium, the population was expanded over four generations to ensure the availability of sufficient individuals for sampling 100 different replicates. Throughout these four expansion generations, each individual was randomly mated with eight different animals, producing one offspring per mating. Consequently, the population size was multiplied by a factor of four in each generation. At the end of this expansion phase, the population comprised 25,600 individuals, serving as the source to sample individuals of the base population (*t *= 0 in the breeding program) for each of the 100 replicates. At this stage (ie, expanded population), there were a total of 54,778 SNPs and 55,185 non-marker loci still polymorphic. The *H_e_* calculated across all segregating loci (SNPs and non-marker loci) was 0.183, while the linkage disequilibrium between consecutive loci, measured as *r^2^* (the squared correlation between pairs of loci), was 0.136.

The individuals sampled from the expanded population served as the founders of the breeding program or base population (50 or 100 individuals in each replicate, depending on the simulated scenario, see below). At that point, a quantitative trait (the target of the subsequent breeding program) was defined with an initial heritability of *h^2^* = 0.4, which could correspond to, for example, growth traits usually selected in many breeding programs. The phenotypic mean (*µ*) and variance (*V_P_*) were set at 0 and 1, respectively. This trait, measured in both sexes, was controlled by 1,000 additive loci (QTL), with no epistatic interactions between them. The additive effect of QTL *i* (*a_i_*) was sampled from a normal distribution with mean 0 and variance $V_{A}/[2p(1-p)n_{\mathrm{sel}}]$, where *V_A_* is the initial additive variance ($h^{2}V_{P}=0.4$), *P* is the average frequency across selective loci and *n_sel_* is the number of selective loci (ie, 1000). Consequently, the expected additive variance summed over all loci equals *V_A_*. The phenotypic value for an individual *j* was obtained as $P_{j}=µ+\sum_{i=1}^{\mathrm{nsel}}x_{i}a_{i}+e_{j}$, where *x_i_* is an indicator variable that takes values 1, 0, or −1 for homozygous 11, heterozygous 10 or homozygous 00, respectively, and *e_i_* is the individual environmental deviation that was sampled from a normal distribution with mean 0 and variance $V_{E}=V_{A}(1-h^{2})/h^{2}$.

One locus was chosen at random to control the expression of a non-lethal monogenic genetic defect among all the loci having a particular allelic frequency. We assumed that the locus was known and its genotype available for all the candidates to selection. Therefore, there was no need to define a gene action, because carriers of the deleterious allele could be detected even if the defect was recessive. This assumption allows the evaluation of allele frequencies because all individuals are genotyped. Three ranges of initial allelic frequencies for the genetic defect were considered to account for different levels of prevalence of the disease in the population: 0.1–0.2, 0.4–0.5, and 0.7–0.8. We coded the deleterious allele as 0 and the neutral allele as 1. At the beginning of the selection process genotypic frequencies corresponded to the Hardy-Weinberg expectations, with the number of homozygous and heterozygous individuals proportional to the allelic frequencies. As the generations went by, the number of carriers of deleterious alleles decreased as a consequence of the management procedure.

### Breeding program

Three different population structures, mimicking a high-prolificacy species breeding program, were examined in this study.

(500/100; evaluated individuals/selected individuals): A hundred founders were randomly mated to generate 50 full-sib families, each producing 10 offspring (half of each sex) genotyped for the locus controlling the defect and measured for the selected trait. From the 500 candidates, 100 individuals (half of each sex) were selected and randomly mated to create the next generation, comprising 50 full-sib families.

(500/50): Fifty founders were randomly mated to create 25 full-sib families, obtaining 20 genotyped and measured offspring (half of each sex) per mating pair. From these 500 individuals evaluated and considered as candidates for selection, 50 individuals (half of each sex) were selected each generation and mated at random to produce 25 full-sib families again.

(1500/100): A hundred founders were randomly mated to also produce 50 full-sib families, but with each family yielding 30 genotyped and measured offspring (half of each sex). This led to a pool of 1500 candidates, from where 100 (half of each sex) were selected and randomly mated to generate again 50 full-sib families. Family sizes of this magnitude are realistic for several high-prolificacy species, particularly in aquaculture breeding programs.

It must be highlighted that differences between scenarios are related to the number of evaluated (ie, candidates to selection) and actually selected individuals. Therefore, different selection differentials (pressures) are achieved corresponding to 20%, 10%, and 7%, respectively. Moreover, considering different numbers of selected individuals allows for simulating different levels of genetic drift, affecting the loss of genetic diversity.

In all scenarios, selection was performed by truncation on the individual values for a combined index (which included the genomic evaluation, see next section) over ten discrete generations. Therefore, at each generation, animals were sorted by their index value and top males and females selected by truncation. Afterwards, they were randomly mated to create the full-sibs families of the next generation.

### Genetic evaluation

Estimated breeding values for the selected trait were obtained through genomic evaluations. Genotypes and phenotypes for all individuals were available. Data from the candidates to selection within a specific generation were used in the evaluation, together with the information (genotype and phenotype) from individuals in all preceding generations. Genomic estimated breeding values (**GEBV**) were calculated through a genomic BLUP_SNP approach, implemented in the GS3 software (Legarra et al. 2011), assuming the following model:


y=1μ+Zu+e

where ***y*** is the vector of phenotypic values, **1** is a vector of 1 s, *µ* is the population mean, **u** is a vector of additive SNP effects, **e** is the vector of random residuals, and **Z** is an incidence matrix constructed from the SNP genotypes for each individual. The element of the *i*^th^ row and *j*^th^ column in **Z** was 2, 0, or 1, if the *i*^th^ individual was, respectively, homozygous 11, heterozygous 10, or homozygous 00 for the SNP *j*. SNP effects were assumed uncorrelated and $V(u)=I\sigma_{u}^{2}$, where **I** is the identity matrix and $\sigma_{u}^{2}$ is the variance of the additive SNP effects. Variance $\sigma_{u}^{2}$ was calculated by dividing the simulated total additive variance by $2\sum \left[p_{i}(1-p_{i})\right]$, where $p_{i}$ is the frequency of allele 1 of SNP *i* in the group of individuals evaluated at *t *= 0 (Fernández et al. 2021). Variance $\sigma_{u}^{2}$ was kept constant across the simulated generations. The **GEBV** for each individual was calculated by adding estimated additive genetic values across the SNPs. For SNP *i*, estimated additive genetic values were equal to $2(1-p_{i})\hat{a_{i}}$, $(1-2p_{i})\hat{a_{i}}$ or $-p_{i}\hat{a_{i}}$ for homozygotes 11, heterozygotes 10, and homozygotes 00, respectively, where ($\hat{a_{i}}$) is the estimated additive SNP effect obtained through the BLUP_SNP. TBV were calculated in the same way but using the real effects and frequencies of QTLs.

To account jointly for the response on the target trait and the prevalence of the genetic defect, a selection index (**SI**) was constructed as


SI=GEBV-GCw

where SI is the vector containing the index value for each individual, GEBV is the vector of their genomic estimated breeding values for the selected trait and GC is a vector with the gene content (Falconer and Mackay 1996) for the genetic defect. Specifically, we consider GC as the number of deleterious alleles carried by a particular individual: two alleles 0 implies gene content equal to 2, one allele 0 implies gene content equal to 1, and two alleles 1 implies gene content equal to 0 (Forneris et al. 2015). Finally, w is the weight given to the target genetic defect. The weights evaluated were 0, 0.01, 0.1, 0.5, and 1. A weight of 0 implies no selection on the genetic defect and, consequently, maximizing the selection pressure (and the expected response) for the productive trait. Contrarily, a weight of 1 is almost equivalent to the strategy where all carriers are discarded (strict culling), which is the benchmark situation regarding the speed of removal of the deleterious alleles.

As a summary, the different scenarios simulated were a combination of the population structure (ie, number of evaluated/selected individuals), the initial frequency of the deleterious allele, and the weight given to the gene content for the locus controlling the defect in the selection index, as can be seen in Table 1.

**Table 1:** The different scenarios simulated as a combination of the population structure (ie, number of evaluated/selected individuals), the initial frequency of the deleterious allele (*P*) and the weight given to the gene content for the locus controlling the defect in the selection index (*w*). ×: scenarios where extra simulations were performed with the correlation between the gene content at the locus controlling the defect and the breeding value for the productive trait above 0.2.

Population structure	Initial frequency	Weight (*w*)				
		0	0.01	0.1	0.5	1
**500/100**	0.1–0.2	*	*	*	*	*
	0.4–0.5	*	*	*	*	*
	0.7–0.8	*	*	*	*	*
**500/50**	0.1–0.2	×	×	×	×	×
	0.4–0.5	×	×	×	×	×
	0.7–0.8	×	×	×	×	×
**1500/100**	0.1–0.2	*	*	*	*	*
	0.4–0.5	*	*	*	*	*
	0.7–0.8	*	*	*	*	*

In each population structure, the first figure is the number of evaluated (candidates) and the second is the number of selected individuals.

### Correlated effects

In an extra scenario, the locus controlling the defect was chosen in such a way that the gene content of each individual was correlated with the breeding value for the productive trait. Particularly, the correlation was forced to be above 0.2. The expectation is that, in this situation, it would be more difficult to remove the deleterious allele while increasing the performance for the selected trait at the same time. These additional simulations were carried out only for the case of 500/50, for all the evaluated initial frequencies of the deleterious allele and all *w*.

### Evaluated parameters

The main comparisons were based on the evolution of the mean TBV (representing the response to selection, or genetic gain), the variance of these TBV (indicative of the potential for future responses to selection), the *H_e_* in the non-marker loci and the genealogical inbreeding (*F*) as measures of the genetic diversity, and the allelic frequency of the genetic defect, marking the effectiveness in the removal of the deleterious allele. Results presented are the averages of 100 replicates. A Fortran 90 code was developed to perform all simulations.

## Results

**Figure 1:**
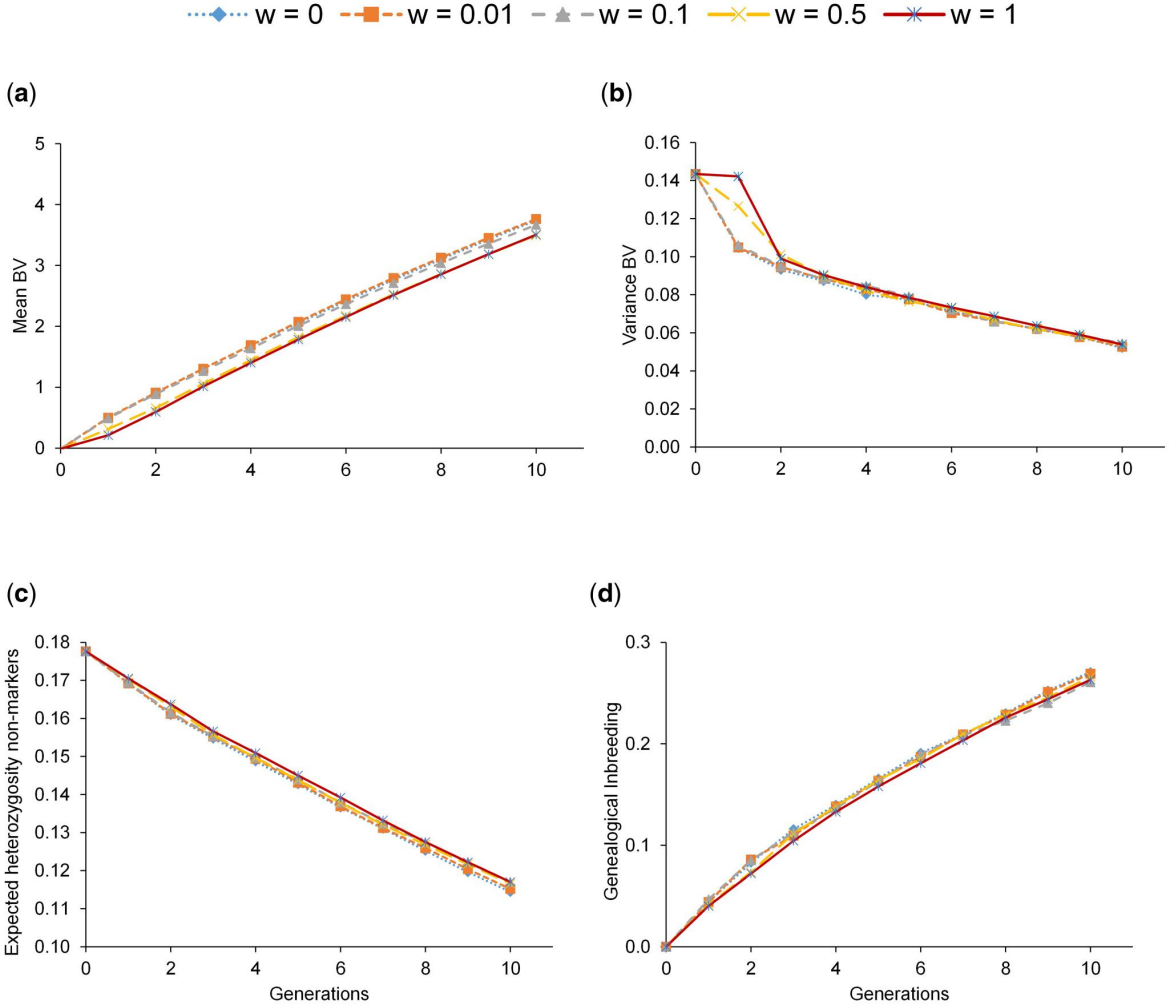
Mean true breeding values (a), variance of the true breeding values (b), expected heterozygosity for non-markers loci (c), and genealogical inbreeding (d) for the scenario with 500 individuals evaluated and 50 individuals selected. The range of initial allele frequency of the deleterious allele was 0.7–0.8. *w* indicates the weight given to the genetic defect in the selection index.

Figure 1 shows the mean true breeding values (TBV), the variance of the TBV, the *H_e_* for non-marker loci, and the genealogical *F* for the scenario comprising 500 individuals’ candidates and 50 selected, initial frequencies between 0.7 and 0.8, and different weights given to the gene content (*w*). Results for the rest of the scenarios (ie, combinations of the number of evaluated/selected individuals and initial frequencies) can be found in Figures S1 and S2. A relevant observation is that there was a steady increase of the mean TBV during the 10 generations of selection, whatever the value of *w* (Figure 1a). Actually, there was almost no effect of the removal strategy but slight differences in the scenarios when the initial prevalence was very high (large *p*), where the response was slower as the value of *w* increased, leading to a marked reduction in genetic gain when *w *= 1 (strict culling), because more individuals are removed, including some with top EBVs. This observation was the same for all breeding schemes, as it is shown in Table 2 for mean TBVs at different generations. Obviously, the largest global responses were obtained in the scenarios with 1,500 candidates and 100 individuals selected, due to the higher selection intensity exerted.

**Table 2:** Mean true breeding value under the three simulated scenarios, the five weights for the genetic defect considered and the three ranges of initial frequencies at different generations (*t*).

Population structure			500/100			500/50			1500/100		
Initial frequency	*w*	*t*	1	2	10	1	2	10	1	2	10
**0.1–0.2**											
	0		0.39	0.73	3.21	0.49	0.91	3.75	0.59	1.07	4.44
	0.01		0.40	0.73	3.20	0.49	0.91	3.71	0.59	1.07	4.43
	0.1		0.39	0.72	3.20	0.49	0.90	3.70	0.58	1.06	4.42
	0.5		0.34	0.68	3.17	0.45	0.88	3.72	0.55	1.03	4.40
	1		0.34	0.67	3.15	0.44	0.87	3.72	0.54	1.03	4.38
**0.4–0.5**											
	0		0.39	0.72	3.19	0.50	0.91	3.74	0.58	1.06	4.43
	0.01		0.40	0.73	3.20	0.50	0.91	3.71	0.58	1.06	4.44
	0.1		0.38	0.71	3.16	0.49	0.90	3.73	0.56	1.04	4.42
	0.5		0.24	0.57	3.06	0.35	0.77	3.62	0.42	0.92	4.34
	1		0.15	0.51	3.02	0.31	0.74	3.59	0.39	0.89	4.30
**0.7–0.8**											
	0		0.39	0.72	3.17	0.50	0.91	3.74	0.59	1.08	4.45
	0.01		0.39	0.72	3.18	0.50	0.91	3.76	0.59	1.07	4.44
	0.1		0.38	0.71	3.13	0.49	0.89	3.67	0.57	1.05	4.37
	0.5		0.24	0.46	2.95	0.32	0.66	3.49	0.37	0.81	4.23
	1		0.19	0.39	2.91	0.21	0.59	3.51	0.18	0.72	4.16

In each population structure, the first figure is the number of evaluated (candidates) and the second is the number of selected individuals. In all cases, TBV at *t *= 0 was zero. Standard error of estimates ranged between 0.07 and 0.08.

As expected, the variance of the breeding values decreases during the 10 generations of selection (Figure 1b), especially in the first generation (Bulmer effect). However, when *w* was large and *P* was also high, the decrease in variance was slower during the early generations. Actually, for *P *= 0.7–0.8 and *w *= 1 there was no drop until the second generation.

The decrease in *H_e_* for non-markers during the 10 generations of selection was barely affected by the initial frequencies of the deleterious allele and the weight given to the defect in the selection index (see Figure 1c and Figures S1 and S2). In parallel, *F* rate was similar within scenarios, whatever the values of *p* and *w* (Figure 1d and Figures S1 and S2). Obviously, values of *H_e_* (*F*) were lower (higher) for scenario 500/50 than for the others, as the number of selected individuals each generation was half and, therefore, the effective population size lower.

**Figure 2:**
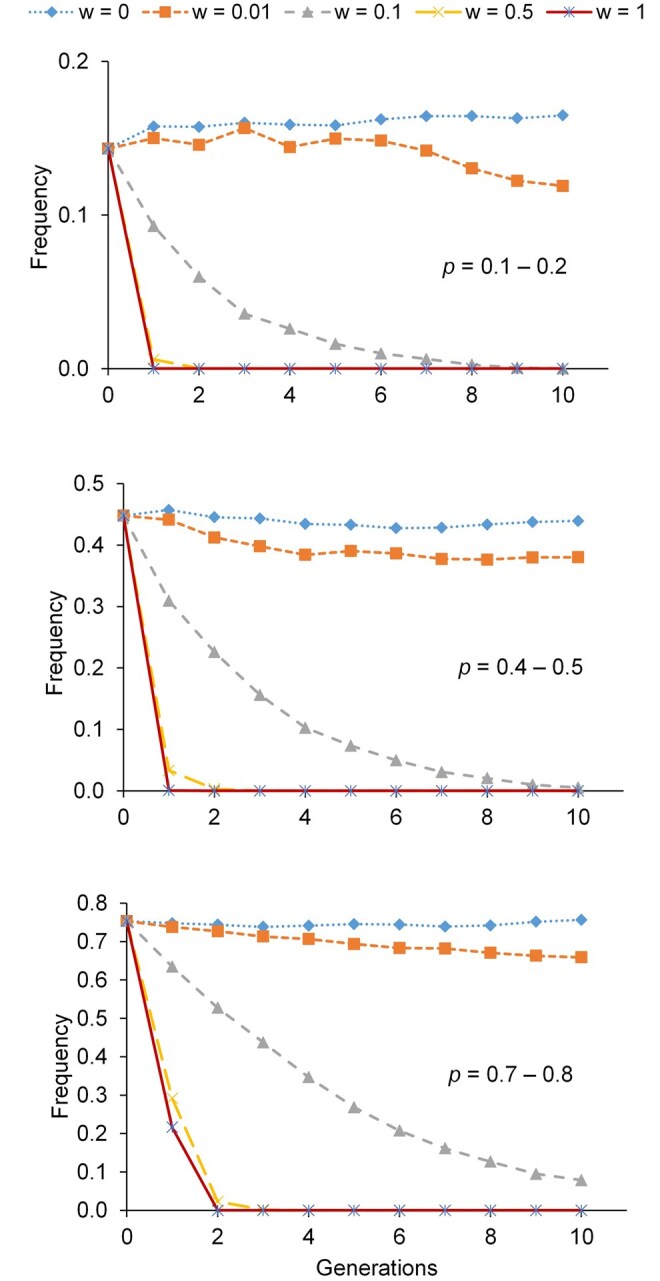
Evolution of the deleterious allele frequencies for the scenario with 500 individuals evaluated and 50 individuals selected. *P* indicates the range of initial allele frequency of the deleterious allele. *w* indicates the weight given to the genetic defect in the selection index.

The allelic frequencies of the simulated genetic defect (Figure 2 and Figures S1 and S2) remained nearly unchanged along the 10 simulated generations when no weight (*w *= 0) was given to the genetic defect (whatever the initial frequency). Notice that in this scenario, no selection occurs on the defect, and, thus, the only change of the frequencies is due to genetic drift. Similarly, with a tiny weight on the genetic defect (*w *= 0.01), the removal was little effective, with high frequencies of the deleterious allele still at *t *= 10. Even with *w *= 0.1 it took eight (10) generations to completely remove the deleterious allele when *P *= 0.1–0.2 (*P *= 0.4–0.5) and still segregating at the end of simulations when *P *= 0.7–0.8. However, for *w *= 0.5 and above, the genetic defect was removed from the population in a maximum of three generations.

When comparing the results in the scenario where an initial correlation between the TBV for the productive trait and the gene count for the deleterious allele existed, no clear differences were found with the scenario with no correlation regarding the mean and the variance of the TBV, *H_e_*, and *F* (Figure S3). The only remarkable observation (in some scenarios) is that the response to selection is slightly lower and the genetic diversity is maintained a bit higher in the correlated scenario (whatever measured through *H_e_* or *F*). Both results were expectable, as in the first generations less individuals with top EBVs (which are on average more related) will be selected, leading to the observed lower response and *F* and the higher *H_e_*.

**Figure 3:**
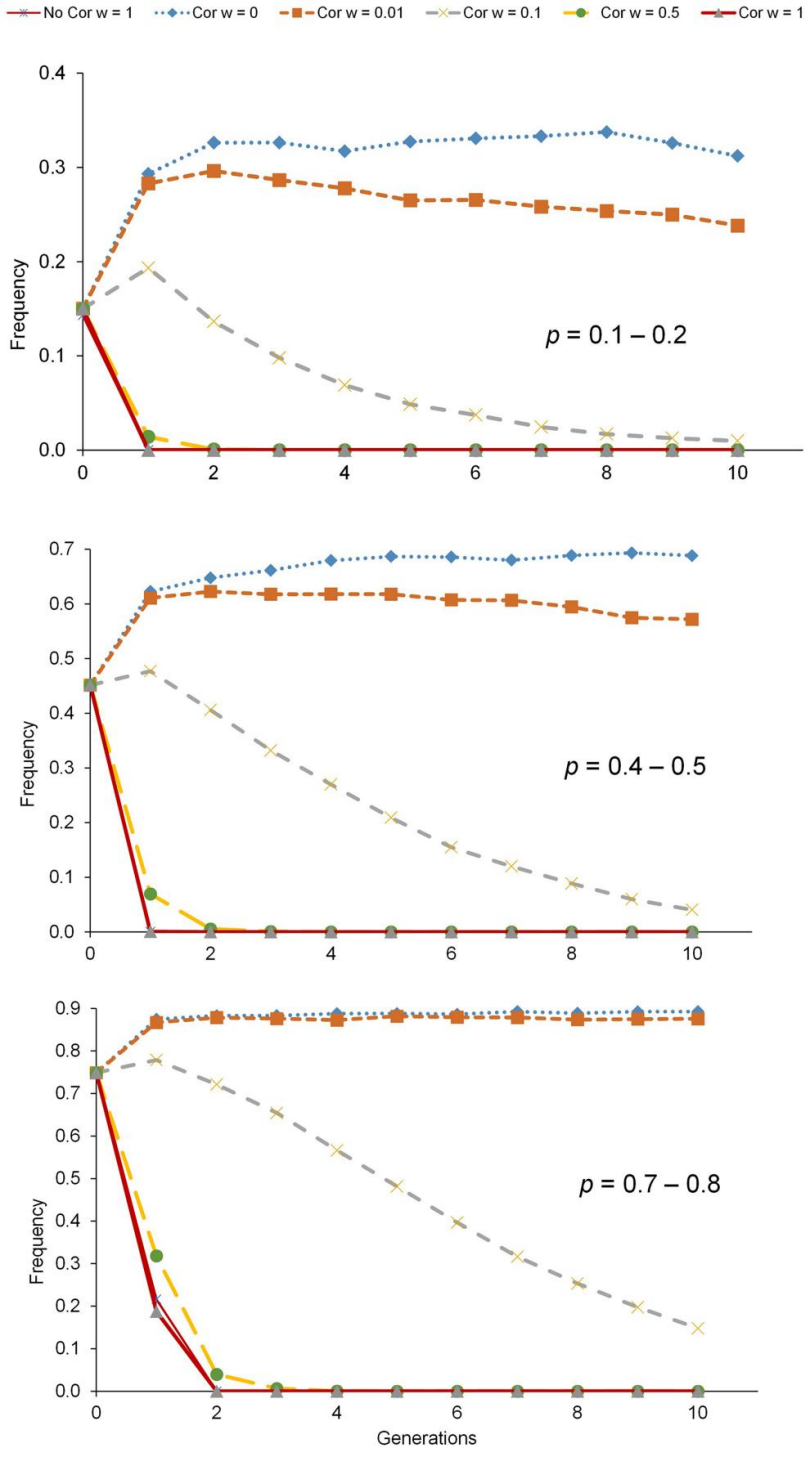
Deleterious allele frequencies for the scenario with 500 individuals evaluated and 50 individuals selected when no correlation (No Cor) or a correlation greater than 0.2 (Cor) between the individual’s breeding value and the gene content for the genetic defect is considered. Different weights (*w*) are shown for the scenario with correlation but only *w *= 1 is shown in the No Cor scenario. *P* indicates the range of initial allele frequency of the deleterious allele.

Regarding the allelic frequency for the genetic defect in this additional scenario with correlation (Figure 3), it must be highlighted that for low *w’*s there is an increase in the frequency of the deleterious allele in the early generations. In fact, for all analyzed values of *P*, the correlation between the additive value and the gene count for the genetic defect makes the deleterious alleles to persist longer in time than when no correlation between them exists.

## Discussion

In this study, the strategy of strict culling of carriers of genetic defects against a balanced selection accounting for the genetic gain and the reduction of the presence of the genetic defect in a high-prolificacy species was compared. The integration of genomic estimated breeding values and weighted gene content within a selection index demonstrated the feasibility of managing the rate at which a genetic defect is eliminated from a population, while simultaneously seeking an optimal balance with genetic gain and diversity. Accounting for the carrier status of an animal can inform breeding decisions aimed at producing the next generation (Sonesson et al. 2003; Berry and Spangler 2023). The simulations were designed to integrate discrete generations and large family sizes, a modeling approach that aligns with population characteristics observed in some animal breeding programs. This scheme/scenario is consistent with the parameters used in previous studies across a variety of species, including pigs (Castro Dias Cuyabano et al. 2019; Zhao et al. 2021; Sharif-Islam et al. 2024) and rabbit populations (Garreau et al. 2005; Blasco et al. 2017). The relevance of such simulation assumptions is also supported in poultry (Mehrabani-Yeganeh et al. 1999; König et al. 2010; Harrison et al. 2023) and aquaculture (Skaarud et al. 2014; Fernández et al. 2021) studies.

Several alternatives have been proposed to decrease the frequency of genetic defects and were tested using simulations or real data from low-prolificacy species (Cole et al. 2025). Some of them adapted selection indices for managing genetic defects using mate allocation schemes (eg, Pryce et al. 2012; Cole 2015; Boichard et al. 2016; Segelke et al. 2016; Bérodier et al. 2021; Hjortø et al. 2021). A breeding strategy that incorporates culling as a pre-selection step (Hjortø et al. 2021) will yield lower genetic progress compared to an index selection approach (Georges et al. 2019). The gene content used in this study associated with a selection index is a gradual and flexible approach, contrasting with the binary logic of strict culling or no action. It also allows breeders to balance genetic gain and genetic defect elimination in a continuous rather than categorical way. High initial allele frequencies were deliberately included to represent worst-case scenarios, and attention was drawn to highlight how the balanced approach behaved in the worst situation, although such high frequencies may be uncommon in real breeding programs. In contrast, low-frequency scenarios are more representative of genetic defects (eg, Cole et al. 2025). Our approach revealed that genetic diversity remained relatively stable across simulated scenarios, although better results were observed for intermediate weight of the genetic defect. As expected, the positive correlation between the genetic defect and the simulated productive trait leads to less genetic gain and more genetic diversity with no significant differences in the elimination of the genetic defect. The positive correlation may increase the frequency of the deleterious allele in the earlier generations due to the hitchhiking effect with the simulated productive trait when the weight given to the genetic defect is low. However, when the spurious correlation disappears, the evolution of the frequency follows the same pattern as in the scenarios with no correlation (eg, it remains stable with *w *= 0). These results emphasize the need to consider genetic architecture, not just the allelic frequency or demographic characteristics, especially in the context of genomic selection.

Management and physiological factors, such as fluctuating ratios of genotyped males and females for the genetic defect, overlapping generations, and reduced family sizes, typical of mammalian species like sheep, may intensify the reduction in genetic gain, thereby limiting the overall success of a breeding program (Raoul et al. 2023). These factors require further investigation to fully understand their implications on selection strategies. Accordingly, in low-prolificacy species, it will be essential to apply the kind of strategies explored in this study, but their effectiveness is expected to be more limited. In contrast, the favorable results observed in this study can be attributed to the high prolificacy with large family sizes simulated. These conditions provide a broad pool of candidates of selection and allow for the exclusion of some of these candidates of selection without weakening selection intensity or compromising genetic diversity. Some aquaculture species may justify exploring even higher prolificacy values than those simulated in the present study.

Although Boichard et al. (2016), considering more traits and genetic defects, suggested that the overall impact on genetic gain may remain limited unless the number or frequency of genetic defects is substantial, this assertion requires further investigation. In particular, the relationships between traits and genetic defects have to be thoroughly evaluated, assuming that connection may be spurious, as happened in one of the scenarios of the present study. However, potential pleiotropic effects, in which alleles coding for the genetic defects directly influence traits of interest (eg, Rupp et al. 2015), should be carefully assessed. A positive effect of the deleterious allele on the productive trait would imply a reduction in the genetic gain when trying to remove the genetic defects. Moreover, it could also impact the maintenance of genetic diversity. Understanding these complex interactions is therefore crucial for designing breeding strategies that maximize genetic gain while preserving genetic diversity in the context of the eradication of defects.

While in this study random mating was implemented in the simulations, it will also be important to consider the role of mating strategies in mitigating the expression of genetic defects. Mating primarily affects inbreeding levels and the probability that recessive alleles are expressed. In this context, recessive alleles may be considered to design mating strategies that avoid pairing carriers, thereby minimizing the probability of producing homozygous offspring expressing genetic defects (Charlier et al. 2008; Derks and Steensma 2021). Fernández et al. (2021) demonstrated that exploiting dominance through targeted matings can reduce the occurrence of deleterious homozygotes, while simply implementing minimum coancestry matings was similarly effective at mitigating the harmful effects of inbreeding.

In this study, selection was performed using truncation selection, which is effective for increasing genetic gain but does not explicitly control inbreeding. It would be advisable to manage inbreeding levels while simultaneously maximizing genetic gain thorough the jointly implementation of optimum contribution selection (OCS) (Sonesson et al. 2003). By incorporating an explicit constraint on coancestry, OCS allows for the direct control of overall genetic diversity, ensuring sustainable long-term genetic gain and minimizing the negative effects of inbreeding on a breeding program.

Strict culling strategies become increasingly penalizing as the initial frequency of the deleterious allele increases, because fewer individuals with top EBVs will be selected. In contrast, balanced strategies allow the retention of individuals with high EBV while progressively reducing the frequency of the genetic defect, particularly in high-prolificacy species. In conclusion, this study shows that, in breeding programs dealing with high-prolificacy species, balanced selection strategies provide an effective compromise between genetic gain, maintenance of genetic diversity, and elimination of genetic defects.

## Supplementary Material

skag100_Supplementary_Data

## Data Availability

Data will be provided from the corresponding author upon reasonable request.

## Abbreviations

Ne: effective population size
QTL: quantitative trait loci
TBV: true breeding values
SNP: single nucleotide polymorphism
m: mutation rate per locus and generation
N: population size
nc: number of chromosomes
nl: number of loci per chromosome
He: expected heterozygosity
t: generation
r2: linkage disequilibrium measured as the squared correlation
h2: heritability
µ: phenotypic mean
VP: phenotypic variance
a: additive effect of QTL
VA: additive variance
p: allele frequency
nsel: number of selective loci
P: phenotypic value
x: indicator variable
e: individual environmental deviation
VE: environmental variance
GEBV: vector of genomic estimated breeding values
y: vector of phenotypic values
u: vector of additive SNP effects
e: vector of random residuals
Z: incidence matrix constructed from the SNP genotypes
I: identity matrix
$\sigma_{u}^{2}$: variance of the additive SNP effects
â: estimated additive SNP effects
SI: vector containing the index value for each individual
GC: vector with the gene content of the genetic defect
w: weight given to the evaluated genetic defect
BV: breeding values
F: genealogical inbreeding
EBV: estimated breeding values
OCS: optimum contribution selection

## References

[skag100-B1] Ben Braiek M , Fabre S, Hozé C, Astruc JM, Moreno-Romieux C. 2021. Identification of homozygous haplotypes carrying putative recessive lethal mutations that compromise fertility traits in French Lacaune dairy sheep. Genet Sel Evol. 53:41. 10.1186/s12711-021-00634-1 33932977 PMC8088666

[skag100-B2] Bengtsson C et al 2023. Mating allocations in Holstein combining genomic information and linear programming optimization at the herd level. J Dairy Sci. 106:3359–3375. 10.3168/jds.2022-22926 37028963

[skag100-B3] Bérodier M et al 2021. Improved dairy cattle mating plans at herd level using genomic information. Animal. 15:100016. 10.1016/j.animal.2020.100016 33516018

[skag100-B4] Berry DP , Spangler ML. 2023. Animal board invited review: practical applications of genomic information in livestock. Animal. 17:100996. 10.1016/j.animal.2023.100996 37820404

[skag100-B5] Berry DP et al 2022. The development of effective ruminant breeding programmes in Ireland from science to practice. Irish J Agric Food Res. 61:38–54. 10.15212/ijafr-2020-0149

[skag100-B6] Blasco A , Martínez-Álvaro M, García ML, Ibáñez-Escriche N, Argente MJ. 2017. Selection for environmental variance of litter size in rabbits. Genet Sel Evol. 49:48. 10.1186/s12711-017-0323-4 28532460 PMC5440956

[skag100-B7] Boichard D et al 2016. Prise en compte des anomalies génétiques en sélection: le cas des bovins. INRA Prod Anim. 29:351–358. 10.20870/productions-animales.2016.29.5.3003

[skag100-B8] Botha M , Petrescu-Mag IV, Hettig A. 2014. Genetic disorders in domestic rabbits. (Oryctolagus Cuniculus). Rabbit Gen. 4:7–47. http://www.rg.bioflux.com.ro

[skag100-B9] Caballero A , Toro MA. 2002. Analysis of genetic diversity for the management of conserved subdivided populations. Con Genet. 3:289–299. 10.1023/A : 1019956205473

[skag100-B10] Castro Dias Cuyabano B , Wackel H, Shin D, Gondro C. 2019. A study of genomic prediction across generations of two Korean pig populations. Animals (Basel). 9:672. 10.3390/ani9090672 31514411 PMC6770396

[skag100-B11] Charlier C et al 2008. Highly effective SNP-based association mapping and management of recessive defects in livestock. Nat Genet. 40:449–454. 10.1038/ng.96 18344998 10.1038/ng.96

[skag100-B12] Cole JB. 2015. A simple strategy for managing many recessive disorders in a dairy cattle breeding program. Genet Sel Evol. 47:94. 10.1186/s12711-015-0174-9 26620491 PMC4666089

[skag100-B13] Cole JB et al 2025. Invited review: management of genetic defects in dairy cattle populations. J Dairy Sci. 108:3045–3067. doi.org/10.3168/jds.2024-26035 39986462

[skag100-B14] Colihueque N , Araneda C. 2014. Appearance traits in fish farming: progress from classical genetics to genomics, providing insight into current and potential genetic improvement. Front Genet. 5:251. 10.3389/fgene.2014.00251 25140172 PMC4121539

[skag100-B15] Derks MFL , Steensma M. 2021. Review: balancing selection for deleterious alleles in livestock. Front Genet. 12:761728. 10.3389/fgene.2021.761728 34925454 10.3389/fgene.2021.761728 PMC8678120

[skag100-B16] Derks MFL et al 2019. Detection of a frameshift deletion in the SPTBN4 gene leads to prevention of severe myopathy and postnatal mortality in pigs. Front Genet. 10:1226. 10.3389/fgene.2019.01226 31850074 PMC6902008

[skag100-B17] Fabre S et al 2021. A novel homozygous nonsense mutation in ITGB4 gene causes epidermolysis bullosa in Mouton Vendéen sheep. Anim Genet. 52:138–139. 10.1111/age.13026 33225458

[skag100-B18] Falconer DS , Mackay TFC. 1996. Introduction to quantitative genetics. Longman.

[skag100-B19] Fernández J , Villanueva B, Toro MA. 2021. Optimum mating designs for exploiting dominance in genomic selection schemes for aquaculture species. Genet Sel Evol. 53:14. 10.1186/s12711-021-00610-9 33568069 PMC7877044

[skag100-B20] Food and Agriculture Organization of the United Nations (FAO). 2013. In vivo conservation of animal genetic resources. First Edition, Rome, Italy.

[skag100-B21] Forneris NS et al 2015. Quality control of genotypes using heritability estimates of gene content at the marker. Genetics. 199:675–681. 10.1534/genetics.114.173559 25567991 PMC4349063

[skag100-B22] Garreau H et al 2005. Gestion et sélection de la souche INRA 1777: Résultats de trois générations de sélection. In: 11 Journées de la Recherche Cunicole, Paris, France.

[skag100-B23] Georges M , Charlier C, Hayes B. 2019. Harnessing genomic information for livestock improvement. Nat Rev Genet. 20:135–156. 10.1038/s41576-018-0082-2 30514919

[skag100-B24] Gozdek M , Mucha S, Prostek A, Sadkowski T. 2024. Selected monogenic genetic diseases in Holstein cattle - a review. Genes (Basel). 15:1052. 10.3390/genes15081052 39202412 PMC11353376

[skag100-B25] Harrison SJ , Siegel PB, Honaker CF, Lewis RM. 2023. Population dynamics of a long-term selection experiment in white Plymouth rock chickens selected for low or high body weight. Poult Sci. 102:102575. 10.1016/j.psj.2023.102575 36907125 PMC10024231

[skag100-B26] Hjortø L et al 2021. Pre-selection against a lethal recessive allele in breeding schemes with optimum-contribution selection or truncation selection. Genet Sel Evol. 53:75. 10.1186/s12711-021-00669-4 34551728 PMC8459560

[skag100-B27] König S et al 2010. Evaluation of inbreeding in laying hens by applying optimum genetic contribution and gene flow theory. Poult Sci. 89:658–667. 10.3382/ps.2009-00543 20308397

[skag100-B28] Legarra A , Ricard A, Filangi O. 2011. GS3: Genomic selection, Gibbs sampling, Gauss-Seidel (and BayesCp); http://snp.toulo use.inra.fr/∼alega rra/manua lgs3_last.pdf/.

[skag100-B29] Lynch M , Walsh B. 1998. Genetics and analysis of quantitative traits. Sinnauer Associates.

[skag100-B30] Mehrabani-Yeganeh H , Gibson JP, Schaeffer LR. 1999. Using recent versus complete pedigree data in genetic evaluation of a closed nucleus broiler line. Poult Sci. 78:937–941. 10.1016/j.psj.2023.102575 10404672

[skag100-B31] Morales-González E , Fernández J, Pong-Wong R, Toro MÁ, Villanueva B. 2021. Changes in allele frequencies when different genomic coancestry matrices are used for maintaining genetic diversity. Genes (Basel). 12:673. 10.3390/genes12050673 33947136 PMC8146037

[skag100-B32] Palhière I et al 2004. Breeding for scrapie resistance in France. In: EAAP Annual Meeting. Bled, Slovenia.

[skag100-B33] Pryce JE , Hayes BJ, Goddard ME. 2012. Novel strategies to minimize progeny inbreeding while maximizing genetic gain using genomic information. J Dairy Sci. 95:377–388. 10.3168/jds.2011-4254 22192217

[skag100-B34] Raoul J et al 2023. Genetic management of cryptorchidism and horn mutations in Manech tête Rouse dairy sheep breed. In: 74th Annual Meeting of the European Federation of Animal Science, Lyon, France.

[skag100-B35] Rupp R et al 2015. A point mutation in suppressor of cytokine signalling 2 (Socs2) increases the susceptibility to inflammation of the mammary gland while associated with higher body weight and size and higher milk production in a sheep model. PLoS Genet. 11:e1005629. 10.1371/journal.pgen.1005629 26658352 PMC4676722

[skag100-B36] Schmidt PI et al 2023. Identification of candidate lethal haplotypes and genomic association with post-natal mortality and reproductive traits in Nellore cattle. Sci Rep. 13:10399. 10.1038/s41598-023-37586-z 37369809 PMC10300016

[skag100-B37] Segelke D , Täubert H, Reinhardt F, Thaller G. 2016. Considering genetic characteristics in German Holstein breeding programs. J Dairy Sci. 99:458–467. 10.3168/jds.2015-9764 26601581

[skag100-B38] Sharif-Islam M et al 2024. Genotyping both live and dead animals to improve post-weaning survival of pigs in breeding programs. Genet Sel Evol. 56:65. 10.1186/s12711-024-00932-4 39294578 PMC11409791

[skag100-B39] Skaarud A , Woolliams JA, Gjøen HM. 2014. Optimising resources and management of genetic variation in fish-breeding schemes with multiple traits. Aquaculture. 420–421:133–138. 10.1016/j.aquaculture.2013.10.033

[skag100-B40] Smith JM , Haigh J. 1974. The hitch-hiking effect of a favourable gene. Genet. Res. 23:23–35. 10.1017/S0016672300014634 4407212

[skag100-B41] Sonesson AK , Janss LL, Meuwissen THE. 2003. Selection against genetic defects in conservation schemes while controlling inbreeding. Genet Sel Evol. 35:353–368. 10.1186/1297-9686-35-5-353 12927071 PMC2697992

[skag100-B42] Zhao Q et al 2021. Long-term impact of conventional and optimal contribution conservation methods on genetic diversity and genetic gain in local pig breeds. Heredity (Edinb). 127:546–553. 10.1038/s41437-021-00484-z 34750534 PMC8626428

