# Supplementary material for: Management of genetic defects in breeding programs of species with high prolificacy
Source: J Anim Sci. 2026 Mar 25;104:skag100. doi: 10.1093/jas/skag100 (PMC13074481; doi:10.1093/jas/skag100)

Supplementary Information

Supplementary Figure 1. Mean true breeding values, the variance of the true breeding values, the expected heterozygosity for non-markers loci, the genealogical inbreeding, and the genetic defect allelic frequency for the scenario with 500 individuals evaluated and 100 individuals selected. *p* indicates the range of initial allele frequency of the deleterious allele of the genetic defect. *w* indicates the weight given to the genetic defect.

Supplementary Figure 2. Mean true breeding values, the variance of the breeding values, the expected heterozygosity for non-markers loci, the genealogical inbreeding, and the genetic defect allelic frequency for the scenario with 1500 individuals evaluated and 100 individuals selected. *p* indicates the range of initial allele frequency of the deleterious allele of the genetic defect. *w* indicates the weight given to the genetic defect.

Supplementary Figure 3. Mean breeding values, the variance of the breeding values, the expected heterozygosity for non-markers, the genealogical inbreeding, and genetic defect allelic frequency for the scenario with 500 individuals evaluated and 50 individuals selected. No correlation (No Cor) or a correlation greater than 0.2 (Cor) between the individuals breeding value and the gene content for the genetic defect at the beginning of the breeding program is considered. Only $w = 1$ is shown. *p* indicates the range of initial allele frequency of the deleterious allele.

Supplementary Figure 1

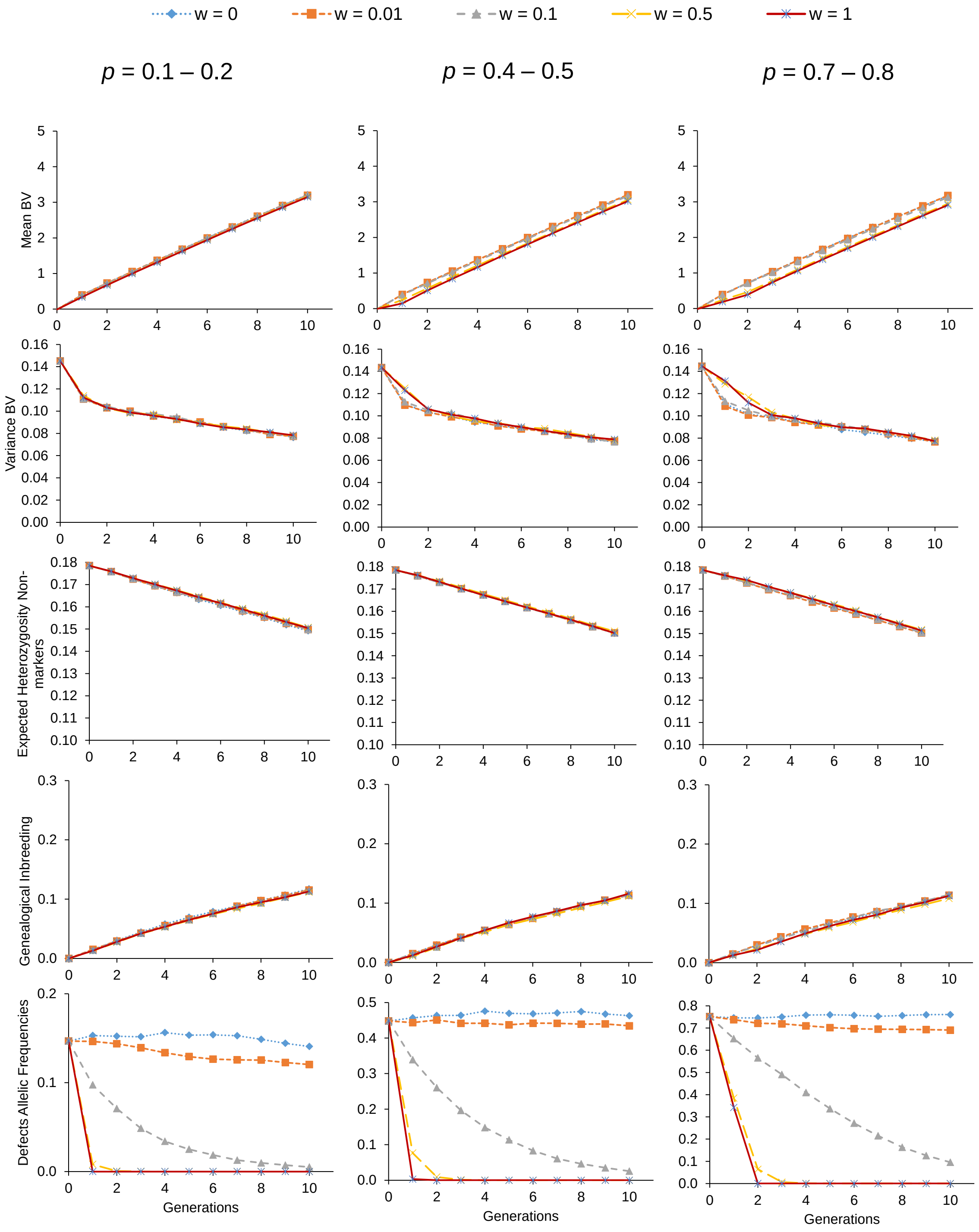

Supplementary Figure 2

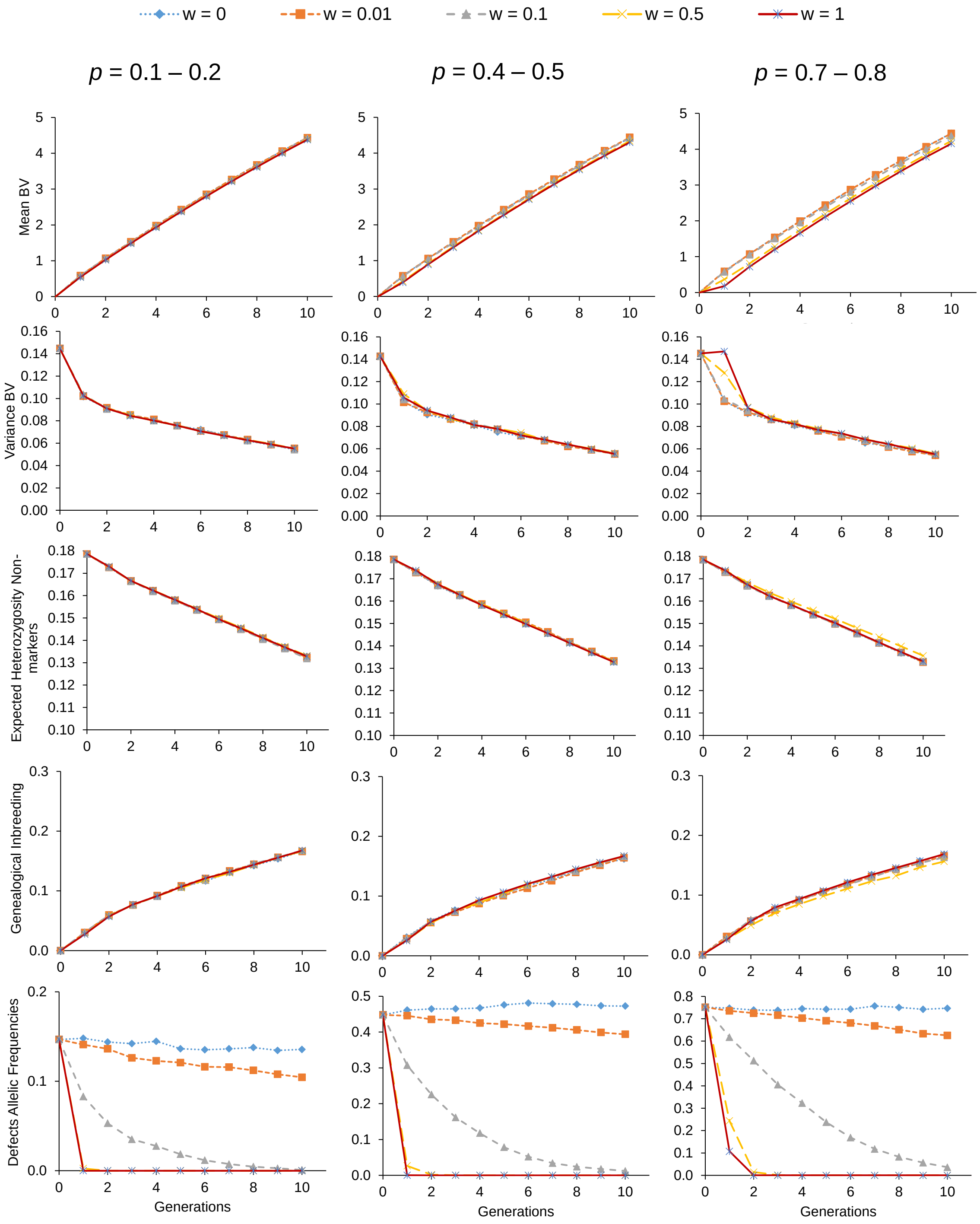

Supplementary Figure 3

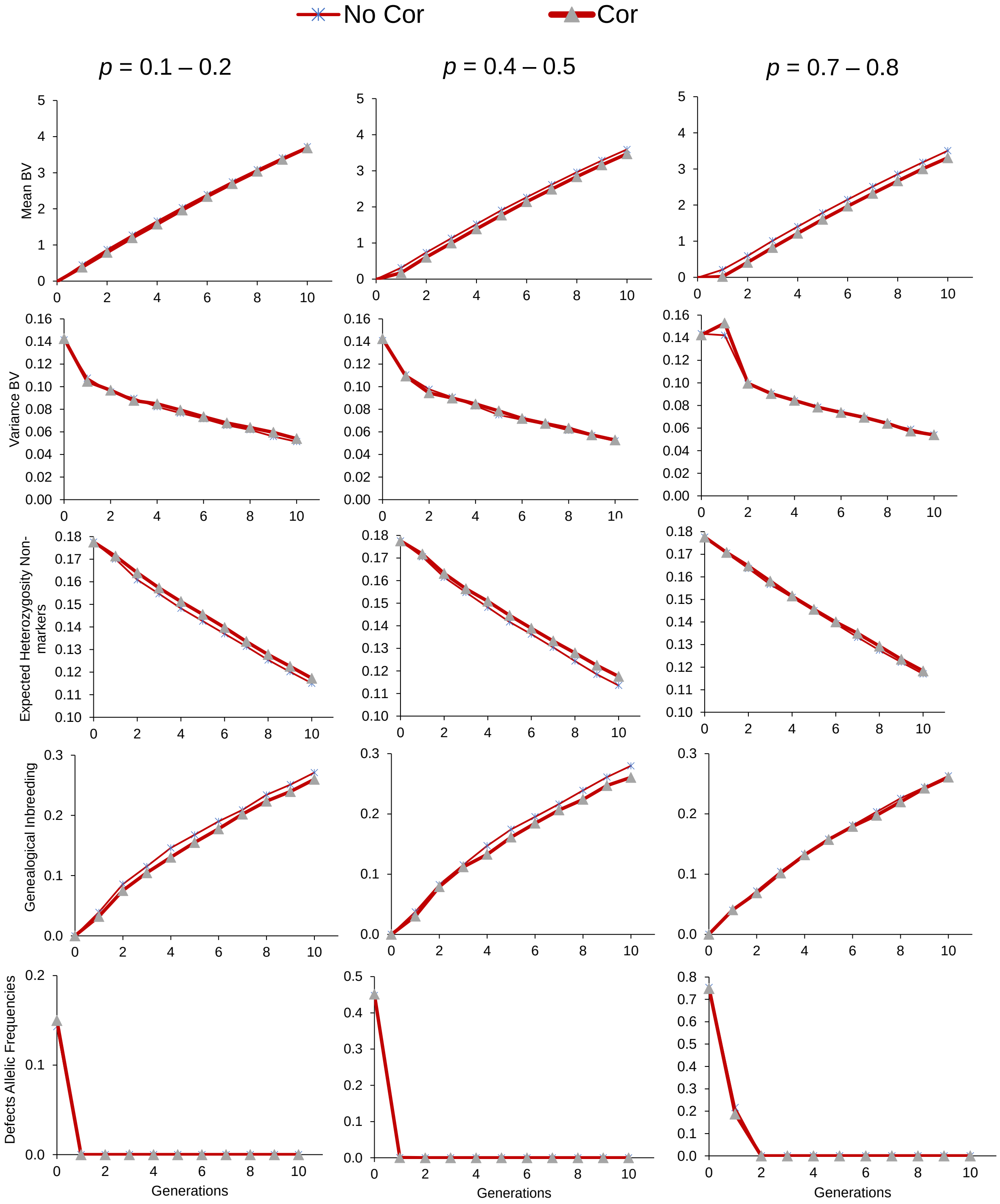

Supplement: skag100_Supplementary_Data [file skag100_supplementary_data.pdf]
